# PCR-RFLP screening of polymorphisms associated with benzimidazole resistance in *Necator americanus* and *Ascaris lumbricoides* from different geographical regions in Brazil

**DOI:** 10.1371/journal.pntd.0006766

**Published:** 2018-09-17

**Authors:** Luciana Werneck Zuccherato, Luis Fernando Furtado, Celi da Silva Medeiros, Carina da Silva Pinheiro, Élida M. Rabelo

**Affiliations:** 1 Departament of Parasitology, Universidade Federal de Minas Gerais, Belo Horizonte, Brazil; 2 Instituto de Ciências da Saúde - ICS, Universidade Federal da Bahia, Salvador, Brazil; McGill University, CANADA

## Abstract

*Ascaris lumbricoides* and *Necator americanus* are soil-transmitted parasites with global geographic distribution, and they represent some of the most common and neglected infections in the world. Periodic treatment with mass drug administration (MDA) in endemic areas is the recommended action put forth by the World Health Organization. However, MDA can cause the selection of subpopulations that possess the genetic ability to overcome the mechanism of drug action. In fact, beta-tubulin gene mutations (codons 167, 198 and 200) are correlated with benzimidazole resistance in nematodes of veterinary importance. It is possible that these SNPs also have strong correlation with treatment resistance in the human geohelminths *A*. *lumbricoides*, *Trichuris trichiura* and hookworms. Here, we aimed to investigate the presence of some of these canonical molecular markers associated with parasite resistance to benzimidazole in *N*. *americanus* and *A*. *lumbricoides* collected from six Brazilian states. Nested-PCR and PCR-RFLP were used to detect mutations at codons 167 and 198 in 601 individual eggs of *A*. *lumbricoides* collected from 62 human stool samples; however, no mutations were found. Codons 198 and 200 were tested in 552 *N*. *americanus* eggs collected from 48 patients using the same methodology, which presented a relative frequency of 1.4% and 1.1%, respectively. The presence of these SNPs in *N*. *americanus* eggs is an important finding, indicating that with high benzimidazole drug pressure there is potential for benzimidazole resistance to be selected in this hookworm. However, at these low frequencies it does not indicate that there is at present any benzimidazole resistance problem. This is the first systematic study performed in South America, and the study yielded a landscape of the genetic variants in the beta-tubulin gene and anthelmintic resistance to soil-transmitted parasites detected by a simple, rapid and affordable genotyping assay of individual eggs.

## Author summary

The soil-transmitted helminths *Ascaris lumbricoides* and the hookworms (*Necator americanus* and *Ancylostoma duodenale*) are the most prevalent intestinal helminths of humans. The standard approach for geohelminth control is large-scale preventive chemotherapy predominantly using benzimidazoles through mass drug administration. Nevertheless, this inexpensive and highly effective strategy can potentially select subpopulations of parasites that become resistant to treatment. In veterinary parasites, intensive reliance on the same anthelmintics has led to the emergence of benzimidazole resistance, but this is not yet proven to helminth parasites of humans. Here we describe a PCR-RFLP approach for single nucleotide polymorphisms (SNPs) detection at codons 167 and 198 in the beta-tubulin gene of *A*. *lumbricoides*, and SNPs at codons 198 and 200 in the beta-tubulin gene of *N*. *americanus* of Brazilian populations. *A*. *lumbricoides* samples showed no mutations in any codons, and mutation at codons 198 and 200 were observed at low frequencies in *N*. *americanus*. The observed data posit important public health issues: the mutations are a substrate for selective pressure and a long-term mass drug administration could allow to the selection of parasites resistant to benzimidazole in Brazil.

## Introduction

Soil-transmitted helminth infections represent important, neglected, tropical diseases that affect approximately one-fourth of the global population. Notably, 820 million people are infected with *Ascaris lumbricoides*, and 439 million people are infected with hookworms (*Necator americanus* or *Ancylostoma duodenale*) [1, 2]. These parasites can cause abdominal pain, colic, diarrhea, nausea, vomiting and, in more severe cases, death of the host. Hookworm infection is the most common cause of iron deficiency anemia and protein-energy malnutrition in children living in developing countries [3]. The standard approach for geohelminth control, including hookworms and *A*. *lumbricoides*, is large-scale preventive chemotherapy predominantly using benzimidazoles through mass drug administration (MDA), based on the drug’s performance in overall reductions in prevalence and reductions in the extent and severity of infection [4]. Nevertheless, this inexpensive and highly effective strategy can potentially select subpopulations of parasites that become resistant to treatment.

Single nucleotide polymorphisms (SNPs) in the beta-tubulin gene at codons 167 (TTC, TTT/Phenylalanine → TAC, TAT/Tyrosine), 198 (GAG, GAA/Glutamic acid → GCG, GCA/Alanine) and 200 (TTC/ Phenylalanine → TAC/Tyrosine) have been linked to benzimidazole resistance in several helminths [5–7]. SNPs at codons 198 [8] and 200 [9, 10] have been reported in hookworms. These common resistance-associated polymorphisms are not frequently found in those populations that did not respond to treatment [9, 11]. Conversely, a mutation at codon 167 was detected at high frequencies in *A*. *lumbricoides* samples [9], while mutations at codon 198 and 200 have never been described for this species.

Brazil is a tropical developing country with high incidence of geohelminths infection. It is estimated that 10,448,507 pre-school-age and school-age children require preventive chemotherapy [12]. MDA has been performed by the government, according to the free attendance health policy guidelines [13]. Nonetheless, there is a lack of studies that have investigated the presence of molecular markers related to parasite resistance to the available drugs. Here, we evaluate the frequency of some of the canonical codons in *N*. *americanus* and *A*. *lumbricoides* that are involved in the process of benzimidazole resistance in specimens from six Brazilian states. To our knowledge, this is the first systematic study performed in South America that examines SNPs present in soil-transmitted helminth human infections associated with benzimidazoles resistance.

## Methods

### Ethical considerations

This work is approved by the Comitê de Ética em Pesquisa—COEP (CAAE 61047216.7.0000.5149) from Universidade Federal de Minas Gerais (UFMG). As we had used human feces obtained from commercial laboratories performing pathology analysis, an informed consent document was not required. We did not obtain any subject identification and the data were analyzed anonymously.

### Parasite material and DNA extraction

Human coproparasitological collection and screening analysis was performed in six Brazilian states. Positive samples for *A*. *lumbricoides* and *N*. *americanus* were stored in 10% formaldehyde for later molecular analysis. The initial isolation of eggs was performed according to Ritchie (1948) with modifications [14]. In summary, 2 ml of stool suspension was homogenized, filtered through gauze and transferred to a 15ml tube. Five ml of sulfuric ether was added and to the suspension and then stirred vigorously, followed by 1 minute centrifugation at 14,000 x g. The supernatant was discarded. Eggs were washed with in a new step by adding 500 μl of 1.0% and 5.0% of hypochlorite for 10 minutes to the *N*. *americanus* and *A*. *lumbricoides* samples, respectively. The material was centrifuged at 14,000 x g, and the supernatant was discarded. The eggs were washed again using 500 μl of ultrapure water, followed by centrifugation at 14,000 x g. The supernatant was then discarded. The pellet was resuspended in 100 μl of ultrapure water for *N*. *americanus*. *A*. *lumbricoides* eggs processing included the additional steps of a) incubation at 30 °C in 500 μl of 0.2 N sulphuric acid for 30 days (for larvae development), b) centrifugation at 14,000 x g and discard of the supernatant, c) washing (resuspension in 500 μl of ultrapure water, centrifugation at 14,000 x g and discard of the supernatant), d) incubation with 500 μl of 1.0% hypochlorite up to the point at which the outerlimiting membrane dissolved using a microscope for visual confirmation, e) repetition of steps a-c followed by the addition of 100 μl of ultrapure water. For DNA extraction, the eggs from both *N*. *americanus* and *A*. *lumbricoides* were observed under an optical microscope, individually pipetted into a volume of 1 μl and transferred to a 500 μl microcentrifuge tube containing 10 μl of buffer, as described by Lake and colleagues [15] and modified by Diawara and colleagues [9]. In total, 864 *A*. *lumbricoides* eggs from 64 patients and 552 *N*. *americanus* eggs from 48 patients, collected in six Brazilian states, were analyzed. None of these samples came from polyparasited patients. Table 1 shows the collection sites, as well as the number of patients and eggs of *A*. *lumbricoides* and *N*. *americanus* collected from each state.

**Table 1:** Collection sites, number of patients and eggs of *A*. *lumbricoides* and *N*. *americanus* used for the SNP genotyping associated with drug resistance.

	*Ascaris lumbricoides*			*Necator americanus*		
	Patients	Total eggs	Eggs per patient (Minimum and maximum)	Patients	Total eggs	Eggs per patient (Minimum and maximum)
**Bahia**	7	70	12–16	6	101	11–21
**Ceará**	16	160	9–15	9	100	7–14
**Maranhão**	13	130	11–16	10	111	9–16
**Minas Gerais**	4	21	9–15	7	80	7–18
**Piauí**	10	100	8–15	10	100	5–12
**Tocantins**	12	120	10–14	6	60	7–12
**Total**	**62**	**601**		**48**	**552**	

### Primer design

All primers were designed using Primer3 (http://bioinfo.ut.ee/primer3-0.4.0/) based on beta-tubulin nucleotide sequences for *N*. *americanus* (EF392851.1) and *A*. *lumbricoides* (EU814697.1) from GenBank and WormBase ParaSite (*N*. *americanus*: PRJNA72135, Assembly GCA_000507365.1; *A*. *lumbricoides*: PRJEB4950, Assembly GCA_000951055.1) (Table 2).

**Table 2:** Primers used for analysis of mutations in the beta-tubulin gene of *A*. *lumbricoides* and *N*. *americanus* and their respective nucleotide changes (when applicable). Positions where changes have been made are underlined.

Species	Primer (5’- 3’)		Change
***N*. *americanus***	***Fa198/200Na***	TTTCCGACACTGTGGTTGAG	
	***Fb198/200Na***	**AATGCTACACTCTCTGTTCACCAGTT	
	***Fm198Na***	ACAGATG**C**GACCTTCTGTATT	A→C
	***Fm200Na***	ACAGATGAGACCT**A**CTGTATT	T→A
	***Rab198/200Na***	GGGAATGGAACCATGTTGAC	
***A*. *lumbricoides***	***AltubF***	ATGTGAGAAAATGCGGTCAT	
	***AltubR***	GGTTGAGGTCTCCGTATGTG	
	***Al167F***	*GCGGTCATAGTTTTCAGGGTTT	
	***Al198R***	**CTCCGTATGTGGGATTTGTAAGC	
	***AlMega167R***	AACAACTGAG**T**ACGAGCTCA	T→A
	***AlMega198F***	ACCGATG**C**AACCTTCTGCAT	A→C

Primers with M13 sequence forward* (CAGGAAACAGCTATGAC) and reverse** (GTAAAACGACGGCCAG) used for wider range in sequencing.

### Wild-type and mutated plasmid constructs

Controls were constructed for the absence (wild-type) and presence of the mutation for each codon for each species. To construct a wild-type control allele for codons 198 and 200 (N198/200Na), PCR amplification was performed with the primers *Fa198/200Na* + *Rab198/200Na* (325 bp) and genomic DNA from *N*. *americanus*. The primers *AltubF* + *AltubR* (596 base pairs, bp) and DNA collected and pooled from *A*. *lumbricoides* eggs were used to construct the wild-type control allele for both codons 167 (N167Al) and 198 (N198Al). PCR amplifications for the wild-type controls were performed using GoTaq Green Master Mix (Promega, USA), with a final concentration of 0.2 μM for each primer, according to the following program: 95°C for 5 min, 30 cycles at 95°C for 30 s, 60°C for 45 s, 72°C for 60 s and a final step of 72°C for 8 min.

To construct the mutated controls for codons 167 (M167Al for *A*. *lumbricoides*), 198 (M198Na for *N*. *americanus*; M198Al for *A*. *lumbricoides*) and 200 (M200Na for *N*. *americanus*), site-directed mutagenesis was performed using the Megaprimer-PCR technique. First, for *N*. *americanus* the wild-type N198/200Na was used as a template for codons 198 and 200 with the primer combinations *Fm198Na* + *Rab198/200Na* (263 bp) and *Fm200Na* + *Rab198/200Na* (263 bp), respectively. For *A*. *lumbricoides*, the wild-type N167Al DNA template was used to perform the megaprimer-PCR for mutated codon 167 with the primers *AlMega167R* + *AltubF* (146 bp), while *AlMega198F* + *AltubR* was used to perform megaprimer for mutated control 198 (94 bp). The *Fm198Na*, *Fm200Na*, *AlMega167R* and *AlMega198F* primers were designed to introduce a mismatch that mimics the mutated sequence corresponding to each codon (Table 2). The site-directed mutagenesis was performed using Kapa HiFi polymerase (Kapa Biosystems, USA) following the manufacturer instructions, and a final concentration of 0.2 μM for each primer, with cycling conditions of 95°C for 3 min, 30 cycles at 98°C for 20 s, 50°C for 45 s, 72°C for 45 s and a final step of 72°C for 8 min. The reaction products were subjected to electrophoresis on 1.0% agarose gels (w/v) (Midsci, USA) stained with GelRed (Biotium, USA). The fragments were excised from the gel, purified (Illustra GFX PCR DNA and Gel Band Purification Kit, GE Healthcare, UK), and measured for concentration. Approximately 20 ng of the first reaction product was used as a megaprimer in combination with 1 μM final concentration of the primers *Fa198/200Na* for mutated M198Na and M200Na (respectively mutated codon 198 and 200, 325 bp each) for *N*. *americanus*, 1 μM of primer *AltubR* and 1 μM of *AltubF* for M167Al and M198Al, respectively (596 pb each), using Kapa HiFi polymerase (Kapa Biosystems, USA) according to the manufacturer instructions with cycling conditions of 95°C for 3 min, 30 cycles at 98°C for 20 s, 60°C for 45 s, 72°C for 45 s and a final step of 72°C for 8 min. The fragments were subsequently cloned using the pGEM-T Easy Vector System (Promega, USA), transformed into XL1-blue cells (Phoneutria, Brazil) and recovered via miniprep (Wizard Plus Miniprep DNA Purification System, Promega, USA). The clones were sequenced, and the absence/presence of the mutations was successfully confirmed.

### Genotyping of individual samples

*In silico* analysis of *A*. *lumbricoides* and *N*. *americanus* beta-tubulin nucleotide sequences retrieved from GenBank and WormBase ParaSite databases were performed using the NEBcutter V2.0 tool (http://www.labtools.us/nebcutter-v2-0/) in order to search for restriction sites that could be used to distinguish between the mutated and the wild type alleles (of 198 and 200 for *N*. *americanus*; and 167 and 198 for *A*. *lumbricoides*) so that a PCR-RFLP approach could be employed. The enzymes *Rsa*I (Promega, USA) and *Bms*I (Thermo Fisher Scientific, USA) were chosen to differentiate between mutated and unmutated codons 167 and 198 of *A*. *lumbricoides*. Sites for enzymes *Alw26*I and *Hpy*AV (Thermo Fisher Scientific, USA) were present in the *N*. *americanus* sequence and therefore used to distinguish between mutated and unmutated alleles for the codons 198 and 200 of *N*. *americanus*, respectively. A suitable enzyme that could differentiate between mutated and unmutated codon 200 of *A*. *lumbricoides* was not found. This same situation was observed for codon 167 of *N*. *americanus*.

For *N*. *americanus*, an initial PCR was performed using one primer pair to amplify the codons 198 and 200 (*Fa198/200Na* + *Rab198/200Na*; 325 bp) using GoTaq Green Master Mix (Promega, USA), with 0.2 μM of each primer and 4.2 μl of the buffer containing the DNA of a single egg. The cycling conditions followed the same parameters as described for the wild-type control synthesis. A semi-nested PCR was performed using 1 μl from the first PCR reaction employing primers *Fb198/200Na* + *Rab198/200Na* (315 pb), using the same PCR conditions as described above. One μl of the products from the second reaction was digested at 37°C for 1 hour with 1 unit of enzyme (*Alw26*I and *Hpy*AV for the codons 198 and 200) in a total volume of 15 μl. The products were subjected to electrophoresis in a 6.0% polyacrylamide gel (w/v) stained with GelRed (Biotium, USA).

For *A*. *lumbricoides*, an initial PCR was performed using one primer pair to amplify the codons 167 and 198 (*AltubF* + *AltuR*; 596 bp). One μl of the PCR product was used as a template for a nested PCR with *Al167F* + *Al98R* (608 pb). The second reaction showed a larger amplicon because the primers had M13 adapters (Table 2). The PCRs and digestions performed for *A*. *lumbricoides* were performed at the same volumes and conditions as used for *N*. *americanus*, except for the enzymes used. The enzymes *Rsa*I and *Bms*I were used for digestion of codons 167 and 198, respectively. The products were subjected to electrophoresis in a 1.5% agarose gel (w/v) stained with GelRed (Biotium, USA). The expected fragment sizes after the digestion of each genotype of each codon are listed in Table 3. Samples with positive PCR-RFLP mutations were sequenced for confirmation.

**Table 3:** Expected sizes of DNA fragments for the three possible genotypes of each codon of the beta-tubulin gene of *A*. *lumbricoides* and *N*. *americanus* after digestion with the appropriate restriction enzymes.

	Codon	Genotype	Fragments (bp)
***A*. *lumbricoides***	**167**	Mutated homozygous (TAC/TAC)	404, 139, 65
		Unmutated homozygous (TTC/TTC)	543, 65
		Heterozygous (TTC/TAC)	543, 404, 139, 65
	**198**	Mutated homozygous (GCA/GCA)	500, 108
		Unmutated homozygous (GAA/GAA)	608
		Heterozygous (GAA/GCA)	608, 500, 108
***N*. *americanus***	**198**	Mutated homozygous (GCG/GCG)	315
		Unmutated homozygous (GAG/GAG)	262, 53
		Heterozygous (GAG/GCG)	315, 262, 53
	**200**	Mutated homozygous (TAC/TAC)	315
		Unmutated homozygous (TTC/TTC)	242, 73
		Heterozygous (TTC/TAC)	315, 242, 73

A negative control sample was included in all amplification runs. Extreme care was taken to avoid contamination with another sample and/or with the control plasmids. Tubes containing the control plasmids were handled in a different room where the DNA samples were manipulated, and filter tips were used in all procedures.

**Fig 1:**
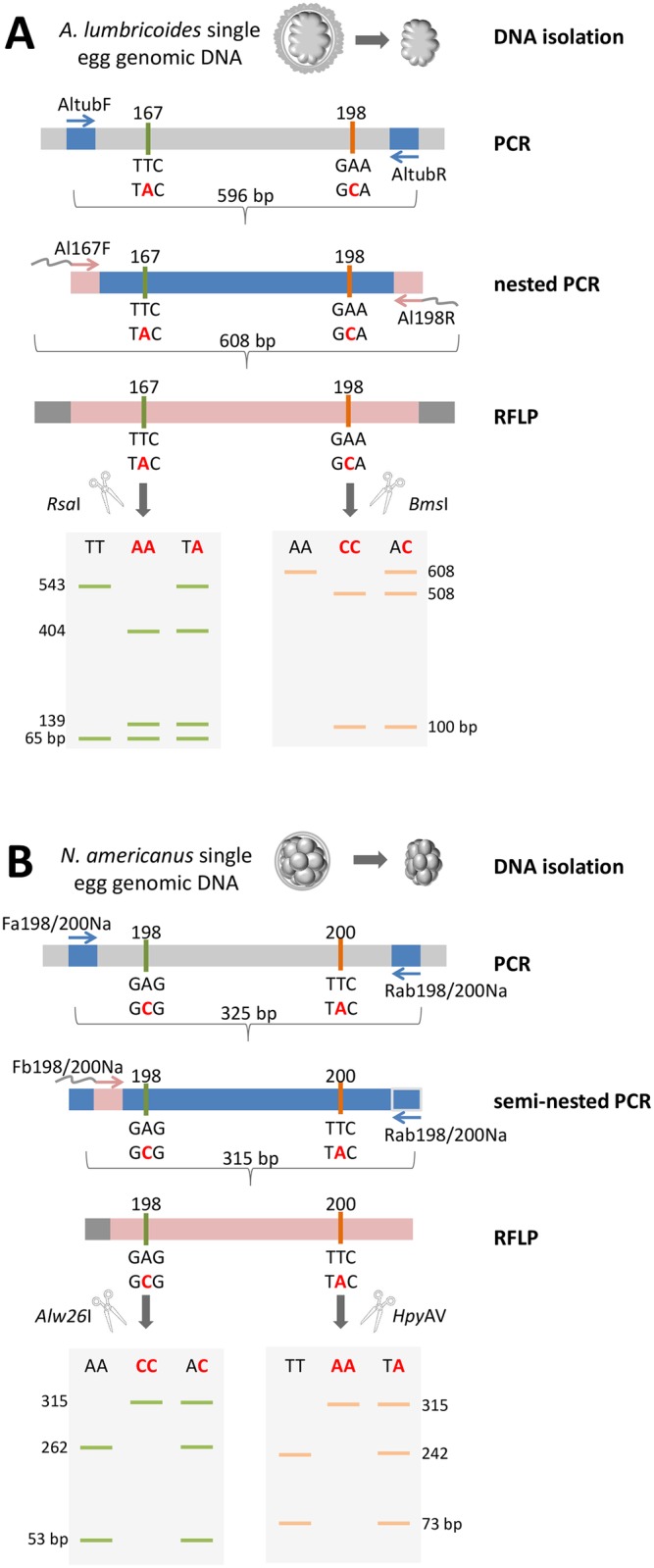
Schematic representation of the PCR-RFLP methodology for single cell screening of beta-tubulin polymorphisms of A) codons 167 and 198 (*A*. *lumbricoides*) and B) codons 198 and 200 (*N*. *americanus*). PCRs were performed using the DNA isolated from a single egg as a template, followed by a nested (for *A*. *lumbricoides*) or semi-nested PCR (*N*. *americanus*). PCR-RFLP was carried out using the selected restrictions enzymes to differ between the mutated and wild-type alleles, as observed by the different sizes of fragments detected in the gel electrophoresis. The gray tails of primers Al167F, Al198R and Fb198/200Na represents the M13 universal primers added to the sequences. The numbers in the gels represent the size of the fragments in base pairs observed after the PCR-RFLP digestion.

Fig 1 shows the methodological scheme adopted for the analysis of all the SNPs in this work.

## Results

We analyzed a total of 1153 geohelminths individual DNA samples from eggs belonging to 110 independent human feces samples collected from six Brazilian states. No mutations were detected in all 601 *A*. *lumbricoides* eggs analyzed for codons 167 and 198 of beta-tubulin gene. Mutation at codon 198 of *N*. *americanus* was observed in three localities: 1) Ceará, with 3.0% positivity (3/100): one homozygous and two heterozygous eggs from the same patient; 2) Minas Gerais, with 3.75% positivity (3/80): one patient had one heterozygous and one homozygous egg (nine eggs evaluated), and another patient had one heterozygous egg (18 eggs analyzed); and 3) Bahia, with 2.0% positivity (2/101): one patient with one homozygous and one heterozygous egg (20 eggs analyzed). Considering all the regions analyzed, 1.4% (8/552) of the eggs examined were found to have the mutation at codon 198.

The tests performed examining codon 200 of *N*. *americanus* showed the presence of the mutation in two states. In Maranhão there was 3.6% (4/111) positivity: one homozygous egg from one patient who had 12 eggs evaluated; another homozygous egg from one patient who had 11 eggs evaluated, and two homozygous eggs from one patient with 10 genotyped eggs. In Bahia there was 2% (2/101) positivity: two heterozygous eggs, one of which was also heterozygous at codon 198, from a patient with 20 eggs evaluated. Therefore, the percentage of examined eggs positive for the mutation at codon 200 was 1.1% (6/552). It is noteworthy that beta-tubulin gene from an egg (from a Bahia patient) was heterozygous for codon 198 and codon 200.

**Fig 2:**
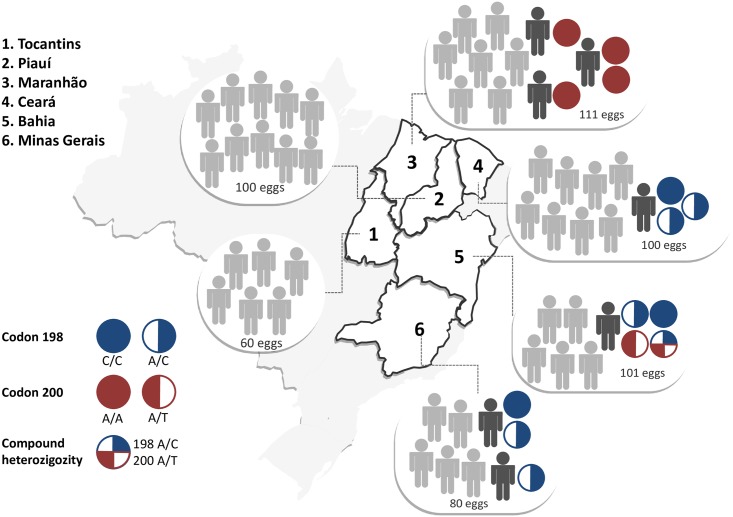
Spectrum of mutations of codons 198 and 200 in 552 *N*. *americanus* samples from six Brazilian regions. Three localities had SNPs at codon 198, representing a 1.4% (8/552) positivity: 1) Ceará with one homozygous and two heterozygous eggs from a same patient (3.0% positivity); 2) Minas Gerais with two heterozygous and one homozygous egg from two patients (3.75% positivity); and 3) Bahia with one homozygous and one heterozygous egg from one patient (2.0% positivity). The overall percentage of egg positivity for the mutation at codon 198 was 1.4% (8/552). For codon 200, the total percentage of egg positivity was 1.1% (6/552), detected in Maranhão (four homozygous eggs from three patients, positivity of 3.6%) and Bahia (two heterozygous eggs from one patient 2.0% of positivity).

S1 Fig shows a representative image of polyacrylamide gel of the PCR-RFLP for *N*. *americanus*. S2 Fig shows a representative image of agarose gel of the PCR-RFLP for *A*. *lumbricoides*. Fig 2 illustrates the genotypes found for codons 198 and 200 in 552 *N*. *americanus* egg samples collected from six Brazilian states.

## Discussion

Treatment with benzimidazoles is the foremost approach for soil-transmitted helminth control; however, the indiscriminate use of these drugs in a target population selects naturally resistant parasites capable of surviving exposure to the drug and can produce resistant offspring [4, 16, 17]. This selective pressure has been associated with the occurrence of SNPs in codons 167, 198 and 200 of the beta-tubulin gene of several helminths [5, 18].

We evaluated the frequency of some of these canonical SNPs in *A*. *lumbricoides* and *N*. *americanus* collected from six Brazilian states by PCR- RFLP technique. Mutations related to resistance to benzimidazoles have been detected by diverse techniques for helminths [10, 19] and fungi [20]. In the present work, a PCR- RFLP molecular screening test was performed to detect mutations in the beta-tubulin gene of *A*. *lumbricoides* and *N*. *americanus*, and controls were synthesized for absence and presence of each SNP analyzed in both species. The PCR- RFLP technique requires the creation or disruption of a restriction endonuclease’s cleavage site by the mutation. In some cases, however, commercially endonucleases are not capable to detect the nucleotide change, as observed for the codons 167 of *N*. *americanus* and 200 of *A*. *lumbricoides*. A wide range of molecular techniques may be employed for the analysis of these SNPs. Methodologies based on qPCR and sequencing have been described for analysis of SNPs in the beta-tubulin gene [19, 21]. Rashwan and colleagues [8] developed a genotyping assay of SNPs on the *N*. *americanus* beta-tubulin gene using the SmartAmp2 method. We therefore aim to overcome this limitation by analyzing these SNPs using ARMS-PCR or tetraprimer ARMS-PCR, as performed by our group for *A*. *caninum* [10, 22, 23].

None of the individual DNA samples of *A*. *lumbricoides* presented mutation at codon 167. This mutation was initially described in *Haemonchus contortus* and later reported in *Teladorsagia circumcincta*, cyathostomes, *Haemonchus placei* and *Trichuris trichiuria* [24]. Notably, a mutation at codon 167 with a distinct elevated frequency was detected in *A*. *lumbricoides* collected from different endemic areas [9]. This finding was not replicated for codon 167 [25] or for any of the three canonical codons in an expanded drug-resistant sampling in Africa [26]. Schwenkenbecher and colleagues [19] detected a very-low-frequency mutation at codon 167 and 200 in hookworms collected from children who received treatment periodically, suggesting that these data may be associated with possible qPCR experimental error. Ishii and colleagues [27] detected a high frequency (61.2%) of this mutation in cyathostomines from Paraná, Brazil. Contrary to the results for most species, polymorphisms at codon 167 were more frequent than mutations at codon 200 in cyathostomes [28].

Analysis of 110 feces samples from patients from Sri Lanka infected with *N*. *americanus* also showed positive results for the mutation at codon 198, but the frequency per helminth was not detected due to the approach of pooling larvae samples instead of searching for SNPs individually [8]. *A*. *caninum* adults and eggs collected from dogs in the United States showed no mutation at codon 198 [29]; similarly, no mutation was observed in a large number of *A*. *caninum* (327 adult worms) obtained from two different states in Brazil [10]. A lack of investigation at codon 198 of the beta-tubulin gene may lead to an underestimation of the reported frequencies. Therefore, it is likely that this mutation has not been extensively described in human hookworms because of the scarcity of available data, rather than being absent in these worms.

The mutation at codon 200 in *N*. *americanus* presented a relative frequency of 1.1% in the Brazilian samples. A frequency of 2.3% of individual eggs analyzed in this species was detected in patients from Kenya; but not detected in samples from Haiti, Panama [9] and Sri Lanka [8]. The highest frequency of this SNP was reported in 36% *N*. *americanus* samples from Haiti where patients had been submitted to MDA [30].

*N*. *americanus* Brazilian population showed homozygous and heterozygous alleles for codons 198 and 200. Curiously, one egg showed mutation in both codons demonstrating compound heterozygosity. None of the samples evaluated in this study exhibited concurrently homozygous mutations at both codons, as observed for other helminths. [31, 32].

Eighty nine eggs of *N*. *americanus* and 163 eggs of *A*. *lumbricoides* did not result in PCR amplifications (results not counted in the sampling number). Three hypotheses might may have accounted for the amplification failure: 1) another mutation (not related to resistance) at the primer’s sequence preventing its annealing and further amplification; 2) sample loss during egg transfer into the lysis buffer; and 3) damage of samples conserved with formaldehyde. The third hypothesis appears to be more likely, since the degradation of DNA in the presence of this preservative has been reported [33, 34].

The medical records of the patients used in the present study were not available, which was a limitation in our analysis. Information regarding the presence of anthelmintic treatment and the drug periodicity would potentially clarify the correlation between SNPs in beta-tubulin gene and benzimidazole resistance in those humans. The absence and low frequency of the selected mutations herein raises the hypothesis that the studied populations did not undergo a mass treatment, or if they did, the periodicity of the chemoprophylaxis was not enough to confer high levels of mutated alleles.

The PCR-RFLP approach, largely used in multi-organism DNA genotyping for decades, has proved to be an affordable, easy and high-throughput screening assay to detect mutations in a substantial number of individual helminths eggs. *A*. *lumbricoides* individual samples showed no mutations at the tested beta-tubulin codons 167 and 198. Low frequencies of mutations at codons 198 and 200 of *N*. *americanus* individual eggs were observed. In a scenario of highly indiscriminate use of albendazoles, the establishment of drug resistance in *N*. *americanus* populations might likely arise. We suggest the inclusion of a comprehensive number of samples encompassing additional geographical regions, to investigate the genetic landscape of those mutations in populations subjected to MDA. The understanding of the genetic components in the dynamics of drug resistance is essential: early monitoring increases the likelihood of delaying the establishment of resistant parasites in the populations.

## Supporting information

S1 Fig Representative PCR-RFLP results from the analysis of 198 (A) and 200 (B) of the *N*. *americanus* beta-tubulin gene using the *Alw*26I and *Hpy*AV enzymes for codons 198 and 200, respectively. Lanes 1 to 3 contain undigested PCR products using the plasmid controls (1: unmutated plasmid, 2: mutated plasmid, and 3: mutated and unmutated plasmid mix). Lanes 4 to 6 contain PCR digestion products using the control plasmids (4: unmutated plasmid, 5: mutated plasmid, and 6: mutated and unmutated plasmid mix). Lanes 7 to 18 contain PCR products using DNA from *N*. *americanus*. Each image is a polyacrylamide gel (6%) that was stained with GelRed (Biotium, USA). MW: 50 bp molecular weight ladder. Expected fragments sizes: codon 198—unmutated: 262 + 53 bp, mutated: 315 bp; and codon 200—unmutated: 242 + 73, mutated: 315 bp.
(TIFF)

S2 Fig Representative PCR-RFLP results from the analysis of codons 167 (A) and 198 (B) of the *A*. *lumbricoides* beta-tubulin gene using the *Rsa*I and *Bms*I enzymes for codons 167 and 198, respectively. Lanes 1 to 3 contain PCR digestion products using the control plasmids (1: mutated plasmid, 2: mutated and unmutated plasmid mix, 3: unmutated plasmid). Lanes 4 to 7 contain PCR products using DNA from *A*. *lumbricoides*. Each image is an agarose gel (1,5%) that was stained with GelRed (Biotium, USA). MW: 100 bp molecular weight ladder. Expected fragments sizes: codon 167—unmutated: 543 + 65 bp, mutated: 404 + 139 + 65 bp; codon 198—unmutated: 608 bp, mutated: 500 + 108 bp.
(TIFF)
