# Supplementary figures and images for: PCR-RFLP screening of polymorphisms associated with benzimidazole resistance in Necator americanus and Ascaris lumbricoides from different geographical regions in Brazil
Source: PLoS Negl Trop Dis. 2018 Sep 17;12(9):e0006766. doi: 10.1371/journal.pntd.0006766 (PMC6141064; doi:10.1371/journal.pntd.0006766)

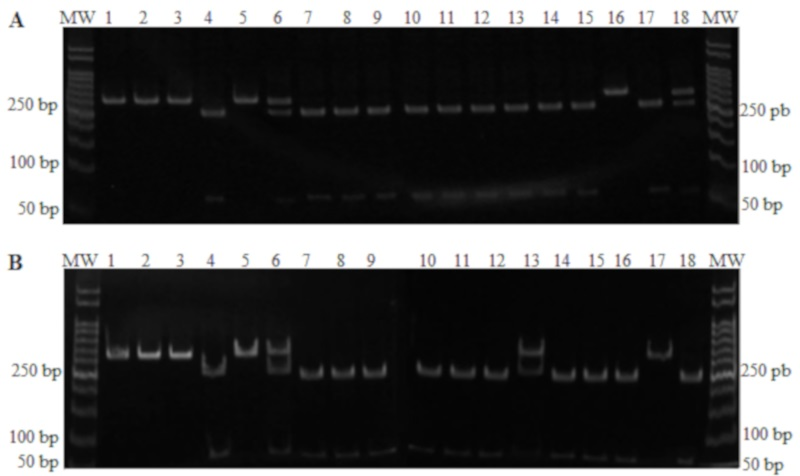

Supplement: S1 Fig — Lanes 1 to 3 contain undigested PCR products using the plasmid controls (1: unmutated plasmid, 2: mutated plasmid, and 3: mutated and unmutated plasmid mix). Lanes 4 to 6 contain PCR digestion products using the control plasmids (4: unmutated plasmid, 5: mutated plasmid, and 6: mutated and unmutated plasmid mix). Lanes 7 to 18 contain PCR products using DNA from N. americanus. Each image is a polyacrylamide gel (6%) that was stained with GelRed (Biotium, USA). MW: 50 bp molecular weight ladder. Expected fragments sizes: codon 198—unmutated: 262 + 53 bp, mutated: 315 bp; and codon 200—unmutated: 242 + 73, mutated: 315 bp. (TIFF) [file pntd.0006766.s001.tiff]

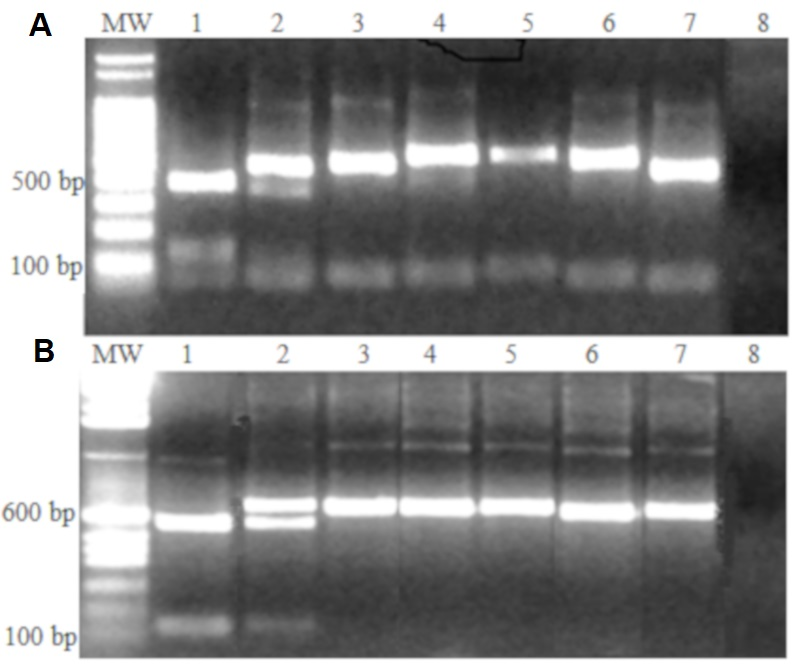

Supplement: S2 Fig — Lanes 1 to 3 contain PCR digestion products using the control plasmids (1: mutated plasmid, 2: mutated and unmutated plasmid mix, 3: unmutated plasmid). Lanes 4 to 7 contain PCR products using DNA from A. lumbricoides. Each image is an agarose gel (1,5%) that was stained with GelRed (Biotium, USA). MW: 100 bp molecular weight ladder. Expected fragments sizes: codon 167—unmutated: 543 + 65 bp, mutated: 404 + 139 + 65 bp; codon 198—unmutated: 608 bp, mutated: 500 + 108 bp. (TIFF) [file pntd.0006766.s002.tiff]
